# Longitudinal tau-PET uptake and atrophy in atypical Alzheimer's disease

**DOI:** 10.1016/j.nicl.2019.101823

**Published:** 2019-04-10

**Authors:** Irene Sintini, Peter R. Martin, Jonathan Graff-Radford, Matthew L. Senjem, Christopher G. Schwarz, Mary M. Machulda, Anthony J. Spychalla, Daniel A. Drubach, David S. Knopman, Ronald C. Petersen, Val J. Lowe, Clifford R. Jack, Keith A. Josephs, Jennifer L. Whitwell

**Affiliations:** a Department of Radiology, Mayo Clinic, Rochester, MN, USA; b Department of Health Science Research (Biostatistics), Mayo Clinic, Rochester, MN, USA; c Department of Neurology, Mayo Clinic, Rochester, MN, USA; d Department of Information Technology, Mayo Clinic, Rochester, MN, USA; e Department of Psychiatry and Psychology, Mayo Clinic, Rochester, MN, USA

**Keywords:** Longitudinal tau-PET, Atrophy, Atypical AD, Multimodal imaging, MCALT, Mayo Clinic Adult Lifespan Template, LPA, logopenic progressive aphasia, PCA, posterior cortical atrophy, ROI, region-of-interest, SCCA, sparse canonical correlation analysis, SUVR, standard uptake value ratio, TBM-SyN, tensor-based morphometry using symmetric normalization

## Abstract

The aims of this study were: to examine regional rates of change in tau-PET uptake and grey matter volume in atypical Alzheimer's disease (AD); to investigate the role of age in such changes; to describe multimodal regional relationships between tau accumulation and atrophy. Thirty atypical AD patients underwent baseline and one-year follow-up MRI, [^18^F]AV-1451 PET and PiB PET. Region- and voxel-level rates of tau accumulation and grey matter atrophy relative to cognitively unimpaired individuals, and the influence of age on such rates, were assessed. Univariate and multivariate analyses were performed between baseline measurements and rates of change, between baseline tau and atrophy, and between the two rates of change. Regional patterns of change in tau and volume differed, with highest rates of tau accumulation in frontal lobe and highest rates of atrophy in temporoparietal regions. Age had a negative effect on disease progression, predominantly on tau, with younger patients having a more rapid accumulation. Baseline tau uptake and regions of tau accumulation were disconnected, with high baseline tau uptake across the cortex correlated with high rates of tau accumulation in frontal and sensorimotor regions. In contrast, baseline volume and atrophy were locally related in the occipitoparietal regions. Higher tau uptake at baseline was locally related to higher rates of atrophy in frontal and occipital lobes. Tau accumulation rates positively correlated with rates of atrophy. In summary, our study showed that tau accumulation and atrophy presented different regional patterns in atypical AD, with tau spreading into the frontal lobes while atrophy remains in temporoparietal and occipital cortex, suggesting a temporal disconnect between protein deposition and neurodegeneration.

## Highlights

• This is the first study investigating longitudinal tau-PET uptake in atypical AD.
• We found that tau spread into the frontal lobes while atrophy remained in temporoparietal and occipital cortex.
• Our results suggest a temporal disconnect between tau deposition and neurodegeneration.

## 1 Introduction

The pathophysiology of Alzheimer's disease (AD) is characterized by neuritic beta-amyloid (Aβ) plaques and tau neurofibrillary tangles (Braak and Braak, 1991; Hyman et al., 2012; Montine et al., 2012). According to the amyloid cascade model, the formation of Aβ plaques triggers the biological events that subsequently cause tauopathy, which is followed by neurodegeneration and, lastly, by the clinical symptoms of dementia (Jack Jr. et al., 2013; Jack et al., 2010; Quiroz et al., 2018). Since AD biomarkers have a presumed temporal sequence, investigating their longitudinal regional changes and associations is paramount to understand the pathogenesis of the disease. While Aβ plaques are deposited relatively uniformly throughout the brain (Cho et al., 2016; Iaccarino et al., 2018; Sepulcre et al., 2016), tau neurofibrillary tangles exhibit characteristic topographical patterns at autopsy (Braak and Braak, 1991) that are thought to reflect the neurodegenerative process (Pontecorvo et al., 2017). Although autopsy represents the gold standard to quantify tau pathology in the brain, in vivo tau-PET imaging using radiotracers like [^18^F]AV-1451 that detect tau pathology (Marquie et al., 2015) allows serial measures of tau over time.

Cross-sectional studies have demonstrated striking [^18^F]AV-1451 uptake in patients with AD, with patterns differing with age (Lowe et al., 2018; Pontecorvo et al., 2017; Tetzloff et al., 2018) and across clinical variants (Ossenkoppele et al., 2016; Scholl et al., 2017). Studies have also shown close spatial relationships between tau-PET uptake and grey matter volume in typical and atypical clinical variants of AD (Dronse et al., 2017; Iaccarino et al., 2018; Ossenkoppele et al., 2016; Sintini et al., 2018; Whitwell et al., 2018; Xia et al., 2017) and demonstrated that tau uptake is also related to antecedent rates of volume loss (Das et al., 2018; Gordon et al., 2018). Two studies have investigated longitudinal regional changes in [^18^F]AV-1451 uptake in AD patients (Harrison et al., 2018; Jack et al., 2018). They show that, as the disease progresses, tau does not accumulate in one area at a time or in a stepwise sequence but its rates of change are observable throughout the brain (Jack et al., 2018) and that tau accumulates longitudinally in regions that have not yet undergone significant atrophy (Harrison et al., 2018). However, these studies focused predominantly on typical Alzheimer's dementia, where the medial temporal lobe is the presumed earliest site of tau deposition. The neurodegenerative process in atypical clinical variants of AD is focused on the neocortex and less is known about how tau deposition and atrophy spread through the brain in these patients.

The aims of this study were to (i) examine regional patterns of change over time in tau-PET uptake and grey matter volume in atypical AD, (ii) investigate the role of age in such longitudinal changes, and (iii) describe the multimodal regional relationships between tau uptake, grey matter volume and their longitudinal rates of change. Our ultimate goal was to increase understanding of the biological processes underlying disease progression in atypical AD.

## 2 Materials and methods

### 2.1 Participants

Thirty atypical AD patients (12 meeting clinical criteria for posterior cortical atrophy (Crutch et al., 2012) (PCA) and 18 meeting clinical criteria for logopenic progressive aphasia (Gorno-Tempini et al., 2008) (LPA)) were recruited from the Mayo Clinic Department of Neurology into an NIH-funded study assessing atypical AD (PI Whitwell) and underwent baseline and one-year follow-up structural MRI, [^18^F]AV-1451 tau-PET scans and Aβ-PET scans. The age range of the patients' cohort was 53–80 years. Patients were excluded from the study if they had a stroke or tumor that could explain their symptoms. All patients were determined to be Aβ positive at baseline Aβ-PET scan. Details of the comprehensive neurological and neuropsychological evaluation can be found in a previous study (Tetzloff et al., 2018). The demographic and clinical features of the cohort are shown in Table 1. Forty-five cognitively unimpaired individuals that had been recruited into the Mayo Clinic Study of Aging (Roberts et al., 2008) and had undergone serial MRI and tau-PET using the same protocol as the atypical AD patients were also included in the study as a control group. Cognitively unimpaired individuals were selected to be Aβ-PET negative. The control cohort consisted of 19 (42%) females (p = 0.01 compared to atypical AD), 13 (29%) APOE e4 carriers (p = 0.61), with median (inter-quartile range) age at baseline scan of 63 years (57–62, p = 0.22). Median Montreal Cognitive Assessment (MoCA) score was 27/30 (27–27) at baseline with median change over time of 0 points (−1, 1). Median scan interval was 489 days (457–520) for MRI and 483 days (430, 508) for tau-PET. The study was approved by the Mayo Clinic IRB, and all patients consented to participate in this study.

**Table 1:** Demographic and clinical features of the patients. Data are shown as median (inter-quartile range), or N (%).

	LPA (*N* = 18)	PCA (*N* = 12)	Total (N = 30)
Demographics			
Female sex	14 (77.8%)	8 (66.7%)	22 (73.3%)
Age at onset (years)	64 (58, 72)	57 (54, 62)	62 (56, 66)
Age at baseline scan (years)	68 (59, 74)	64 (60, 69)	66 (59, 71)
Disease duration (years)	2 (2, 3)	4 (4, 6)	3 (2, 5)
Left handedness	4 (22.2%)	2 (16.7%)	6 (20.0%)
Baseline global PiB	2.49 (2.25, 2.92)	2.49 (2.34, 2.56)	2.49 (2.32, 2.75)
ApoE e4 carrier prevalence	6 (33.3%)	5 (41.7%)	11 (36.7%)
White matter hyperintensity volume at baseline (cm^3^)	12.3 (9.8, 18.3)	16.8 (13.2, 25.1)	13.9 (9.9, 19.7)
Scan interval (days, baseline and follow-up) – MRI	364 (348, 376)	384 (356, 406)	368 (348, 398)
Scan interval (days, baseline and follow-up) – Tau-PET	362 (349, 374)	384 (356, 406)	367 (349, 405)
Scan interval (days, baseline) – MRI and Tau-PET	0 (0, 1)	0 (−1, 1)	0 (0, 1)
Scan interval (days, follow-up) – MRI and Tau-PET	1 (0, 1)	0 (−1, 1)	0 (−1, 1)

Neurological evaluation			
MoCA			
Baseline	19 (17, 22)	18 (15, 25)	18 (16, 24)
Follow-up	14 (11, 17)	13 (10, 18)	13 (10, 18)
Annualized change	−5 (−7, −4)	-6 (−6, −5)	-5 (−7, −4)

Cambridge behavioral inventory			
Baseline	16 (13, 26)	60 (17, 76)	21 (14, 59)
Follow-up	26 (20, 47)	56 (32, 90)	34 (20, 62)
Annualized change	12 (3, 22)	15 (0, 23)	12 (0, 23)

CDR sum of boxes			
Baseline	2 (1, 3)	4 (3, 6)	2 (1, 4)
Follow-up	4 (2, 4)	6 (4, 8)	4 (3, 6)
Annualized change	2 (1, 3)	2 (1, 3)	2 (1, 3)
Optic ataxia			
Baseline	0 (0.0%)	4 (33.3%)	4 (13.3%)
Follow-up	0 (0.0%)	6 (60.0%)	6 (22.2%)

Oculomotor apraxia			
Baseline	0 (0.0%)	4 (33.3%)	4 (13.3%)
Follow-up	1 (5.9%)	5 (50.0%)	6 (22.2%)

WAB Praxis			
Baseline	58 (56, 60)	60 (56, 60)	60 (56, 60)
Follow-up	58 (57, 59)	58 (47, 59)	58 (55, 59)
Annualized change	−2 (−3, 0)	−1 (−2, 0)	−1 (−3, 0)

Gerstmann syndrome (out of 7)			
Baseline	5 (5, 7)	5 (2, 6)	5 (4, 6)
Follow-up	3 (2, 4)	4 (1, 5)	3 (2, 5)
Annualized change	−2 (−3, −1)	−1 (−2, 0)	−2 (−2, 0)

Simultanagnosia (out of 20)			
Baseline	18 (17, 20)	8 (4, 13)	17 (9, 19)
Follow-up	18 (16, 18)	5 (2, 8)	16 (7, 18)
Annualized change	−2 (−2, 0)	−3 (−5, 0)	−2 (−3, 0)

Neuropsychological evaluation			
WMS III VR % ret. MOANS			
Baseline	9 (7, 11)	8 (7, 9)	9 (7, 11)
Follow-up	8 (5, 12)	8 (3, 11)	8 (4, 12)
Annualized change	−1 (−3, 1)	−1 (−4, 3)	−1 (−4, 1)

BDAE sentence repetition			
Baseline	7 (6, 8)	8 (7, 9)	8 (6, 9)
Follow-up	6 (4, 7)	8 (6, 10)	6 (5, 8)
Annualized change	−1 (−2, −1)	0 (−1, 0)	−1 (−2, 0)

Boston naming test			
Baseline	11 (9, 12)	10 (8, 13)	11 (8, 13)
Follow-up	7 (2, 12)	10 (6, 13)	8 (4, 12)
Annualized change	−2 (−4, −1)	−1 (−2, −1)	−2 (−3, −1)

Letter fluency (sum FAS)			
Baseline	26 (21, 32)	34 (28, 47)	31 (22, 36)
Follow-up	18 (12, 25)	29 (20, 42)	22 (14, 32)
Annualized change	−7 (−12, −6)	−9 (−11, −4)	−8 (−12, −6)

Animal fluency			
Baseline	10 (8, 13)	11 (8, 18)	10 (8, 15)
Follow-up	8 (7, 9)	10 (7, 14)	8 (7, 12)
Annualized change	−2 (−4, −1)	−3 (−4, −1)	−2 (−4, −1)

VOSP letters			
Baseline	19 (18, 20)	14 (6, 18)	18 (14, 19)
Follow-up	19 (18, 20)	11 (6, 16)	18 (11, 19)
Annualized change	0 (−1, 0)	−3 (−8, 0)	0 (−3, 0)

Rey-O MOANS			
Baseline	6 (2, 9)	2 (2, 2)	2 (2, 6)
Follow-up	3 (2, 6)	2 (2, 2)	2 (2, 4)
Annualized change	0 (−2, 0)	0 (0, 0)	0 (−1, 0)

MoCA = Montreal Cognitive Assessment Battery; CDR = Clinical Dementia Rating Scale; NPI-Q = Neuropsychiatric Inventory brief questionnaire version; MDS-UPDRS III = Movement Disorder's Society sponsored revision of the Unified Parkinson's Disease Rating Scale; WAB Praxis = Western Aphasia Battery ideomotor apraxia scale; WMS III VR % ret. = Wechsler Memory Scale-III visual reproduction percent retention; BDAE = Boston Diagnostic Aphasia Examination; MOANS = Mayo Older American Normative scale; VOSP = Visual Object and Space Perception Battery; Rey-O = Rey Osterrieth. Details of how optic ataxia, oculomotor apraxia, Gerstmann syndrome and simultanagnosia were assessed are provided in (Tetzloff et al., 2018). Specifically, the simultanagnosia test was designed to assess the individuals ability to perceive the overall meaning/shape of the figure/object/picture instead of recognizing bits and pieces, and included, for example, pictures of overlapping line drawings, pictures of fragmented numbers, and pictures of objects/letters whose shape was created from smaller items.

### 2.2 Image acquisition

All PET scans were acquired using a PET/CT scanner (GE Healthcare, Milwaukee, Wisconsin) operating in 3D mode. For tau-PET, an intravenous bolus injection of approximately 370 MBq (range 333–407 MBq) of [^18^F]AV-1451 was administered, followed by a 20 minute PET acquisition performed 80 min after injection. For Aβ-PET, participants were injected with Pittsburgh Compound B (PiB) of approximately 628 MBq (range, 385–723 MBq) and, after a 40–60 minute uptake period, a 20 minute PiB scan was obtained. Both PiB and [^18^F]AV-1451 scans consisted of four 5-minute dynamic frames following a low dose CT transmission scan. Standard corrections were applied. Emission data were reconstructed into a 256 × 256 matrix with a 30-cm FOV (in-plane pixel size = 1.0 mm, slice thickness = 1.96 mm). All participants underwent a 3 T head MRI protocol that included a magnetization prepared rapid gradient echo (MPRAGE) sequence (TR/TE/TI, 2300/3/900 ms; flip angle 8°, 26-cm field of view (FOV); 256 × 256 in-plane matrix with a phase FOV of 0.94, and slice thickness of 1.2 mm (Jack Jr. et al., 2008) and a fluid-attenuated inversion recovery (FLAIR) (TR/TE = 11,000/147 ms; 22-cm FOV; slice thickness = 3.6 mm) sequence. White matter hyperintensities were segmented and manually edited on the FLAIR images by a trained image analyst using a semi-automated method (Table 1) (Raz et al., 2013).

### 2.3 Image processing

Each tau-PET image was rigidly registered to its corresponding MPRAGE using SPM12. Using ANTs (Avants et al., 2008), the Mayo Clinic Adult Lifespan Template (MCALT) (https://www.nitrc.org/projects/mcalt/) atlases were propagated to the native MPRAGE space and used to calculate regional PET values in the grey and white matter. Tissue probabilities were determined for each MPRAGE using Unified Segmentation (Ashburner and Friston, 2005) in SPM12 (Wellcome Trust Centre for Neuroimaging, London, UK), with MCALT tissue priors and settings (Schwarz et al., 2017). Eighty-four regions-of-interest (ROIs) in the frontal, sensorimotor, temporal, parietal and occipital lobes were selected and the median tau-PET value in each ROI was divided by median uptake in cerebellar crus grey matter to create standard uptake value ratios (SUVR). PET images were not partial volume corrected; however the adopted approach of masking atlas regions based on the segmentation avoids outlying voxels that are mostly non-tissue, and thus reduces the effects of partial volume. Annualized rates of tau accumulation were calculated in each selected ROI as the difference between the follow-up SUVR and the baseline SUVR, divided by the year difference between the two measurements (Chiotis et al., 2017; Jack et al., 2018). Grey matter volume was calculated in same set of 84 ROIs and the values were normalized with respect to each subject's total intracranial volume. Annualized rates of grey matter volume loss were estimated with an in-house developed version of tensor-based morphometry using symmetric normalization (TBM-SyN). The baseline and follow-up MPRAGE images of each subject were co-registered to their common mean with a 9 degree-of-freedom linear registration, and an in-house developed implementation of differential bias correction was run on each subject's scans in order to remove intensity inhomogeneity bias across each subject's serial set of scans. ANTs software was then used to compute a SyN deformation between each scan pair. For each scan pair, we computed and applied the SyN deformation from the late to the early image, and vice-versa, and averaged the deformed image with the stationary image to generate “synthetic” early and late images. We also saved the image log of the determinant of the Jacobian for the deformations, and divided them by the number of days between scans and multiplied by 365.25 to get an annualized log Jacobian image (Vemuri et al., 2015). After applying the Unified Segmentation to the “synthetic” early and late images, mean annualized log Jacobian values (which can be thought as annualized percent change in grey matter volume) were calculated in each ROI. Aβ-PET images were processed similarly to the tau-PET images and a global Aβ-SUVR was generated for each patient, using a cut-point of 1.42 to establish Aβ positivity (Jack et al., 2017). PET and grey matter MR images of each subject were subsequently spatially normalized to the MCALT template and blurred with a 6 and 8 mm full width at half maximum kernel, respectively, for the voxel-wise analyses.

### 2.4 Statistical analyses

#### 2.4.1 Bayesian hierarchical models

To investigate the regional annualized change in tau SUVR (i.e. tau accumulation) and in grey matter volume (i.e. atrophy) in atypical AD relative to cognitively unimpaired individuals, we used two Bayesian hierarchical models, which solve the problem of multiple comparisons while stabilizing estimates across regions and reducing data artifacts (Gelman et al., 2013; Greenland, 2000). The models predicted annualized regional change in atypical AD patients and cognitively unimpaired individuals, with regional random intercepts, random regional baseline age effects (centered at 65, which was the median age in our patients' cohort, and scaled by decade), and a random error term. Groups of random effects for both intercepts and age effects, independently in AD patients and cognitively unimpaired individuals, were assumed to come from normal distributions, with hyperparameters for the mean and variance following a standard normal and half-standard normal distribution, respectively. Results were based on two hundred parallel Markov chain Monte Carlo simulations of length 80,000 thinned to every 40th value each with 15,000 burn in discarded. Each chain had distinct starting points, and results were not sensitive to the choice of the prior distribution. In both models the Gelman and Rubin statistic was approximately one, a good indication of the model fit. To obtain estimates for the atypical AD cohort, a weighted average of the LPA and PCA effects in each ROI was calculated. To compare the lobe-wise annualized change within each modality, we summarised the proportion of the posterior simulations where one lobe-wise average annualized change was greater than another. These analyses were performed in *R* version 3.4.2 (http://www.r-project.org/), using the rjags package (Plummer et al., 2016).

#### 2.4.2 Voxel-based analyses

SPM12 was used to perform multiple regression analyses that assessed differences in tau-PET uptake, MRI grey matter volume and Aβ-PET uptake at baseline and at follow-up in the patients' population relative to the cognitively unimpaired, with age as covariate. To assess longitudinal annualized rates of change in tau SUVR (i.e. tau accumulation) and in grey matter volume (i.e. atrophy) in patients relative to cognitively unimpaired individuals, SPM multiple regression analyses were performed on tau-PET annualized change maps, images created by subtracting the baseline tau-PET image from the follow-up tau-PET image, and dividing by the time difference in years, and on the MRI annualized log Jacobian maps. These analyses were performed for the entire atypical AD group, and separately for the PCA and LPA group. The effect of the patients' age on tau accumulation and atrophy was assessed with SPM one-sample *t*-tests on the patients' tau-PET annualized change maps and MRI annualized log Jacobian maps, with age as covariate. The age effect was assessed only for the entire atypical AD cohort.

#### 2.4.3 Multimodal analyses

Partial Pearson's correlations were performed to assess ROI-level relationships between 1) tau SUVR at baseline and tau SUVR annualized changes, 2) MRI volume at baseline and MRI annualized log Jacobians, 3) tau SUVR at baseline and MRI annualized log Jacobians, 4) tau SUVR annualized changes and MRI annualized log Jacobians. A permutation approach was implemented to correct for multiple comparisons (Avants et al., 2010). These analyses were conducted using Matlab2018a (The Mathworks, The Mathworks, Inc., Natick, MA, USA). Sparse canonical correlation analysis (SCCA) was applied using the PMA (Penalized Multivariate Analysis) *R* package (Witten et al., 2009), to investigate multivariate relationships between the same quantities. Canonical correlation analysis seeks linear combinations of the variables in two datasets that are maximally correlated with each other (Hotelling, 1936). In each analysis, a lasso penalty of 0.2 was assigned to both datasets to achieve the desired level of sparsity (Adams et al., 2019). To display the results, we color-coded the MCALT atlas, i.e. the voxels inside the ROIs that were associated to a non-zero canonical weight were colored. The univariate and multivariate analyses were performed using the entire group of atypical AD subjects; they were not repeated in the separate diagnostic groups due to the small number of subjects in each group.

## 3 Results

### 3.1 Bayesian hierarchical model

**Fig. 1:**
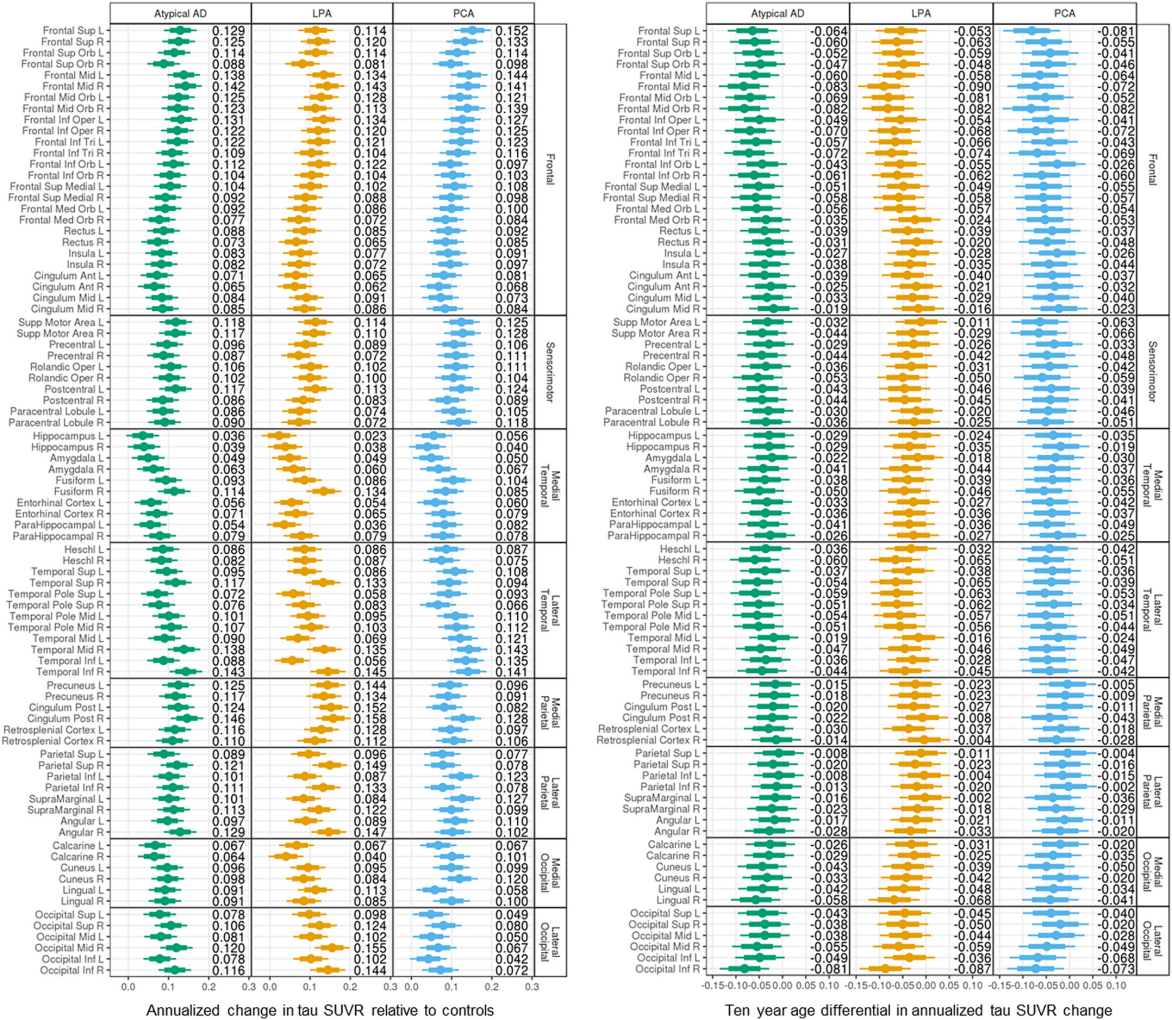
Median and distribution estimates of regional annualized changes in tau SUVR. Bars cover 80% (thick bar) to 95% (thin bar) intervals of posterior estimates (i.e. confidence intervals). Regional decade effects (i.e. how the regional change relates to age) are showed on the right.

**Fig. 2:**
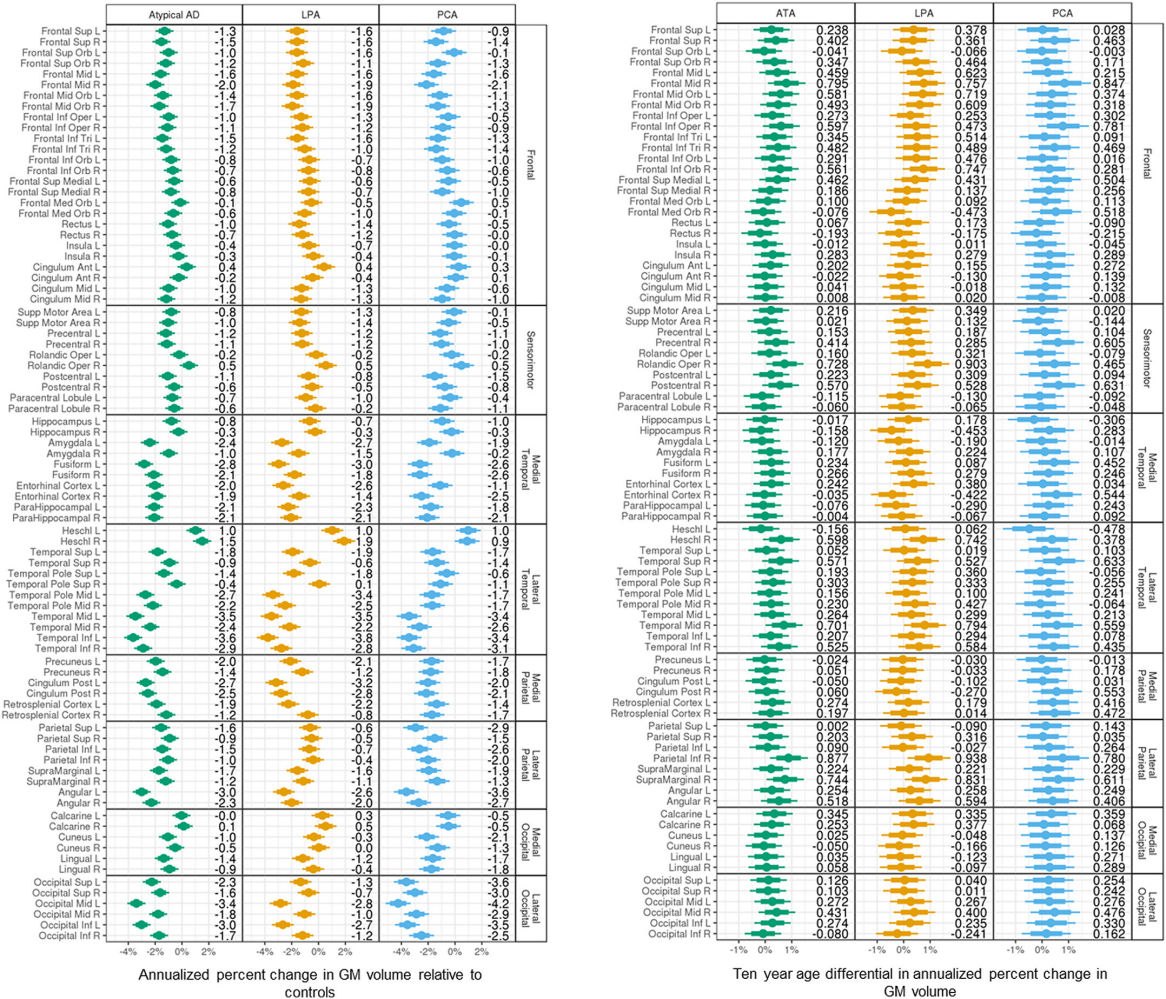
Median and distribution estimates of regional annualized percent changes in MRI grey matter (GM) volumes. Bars cover 80% (thick bar) to 95% (thin bar) intervals of posterior estimates (i.e. confidence intervals). Regional decade effects (i.e. how the regional change relates to age) are showed on the right.

**Supplemental Fig. 1:**
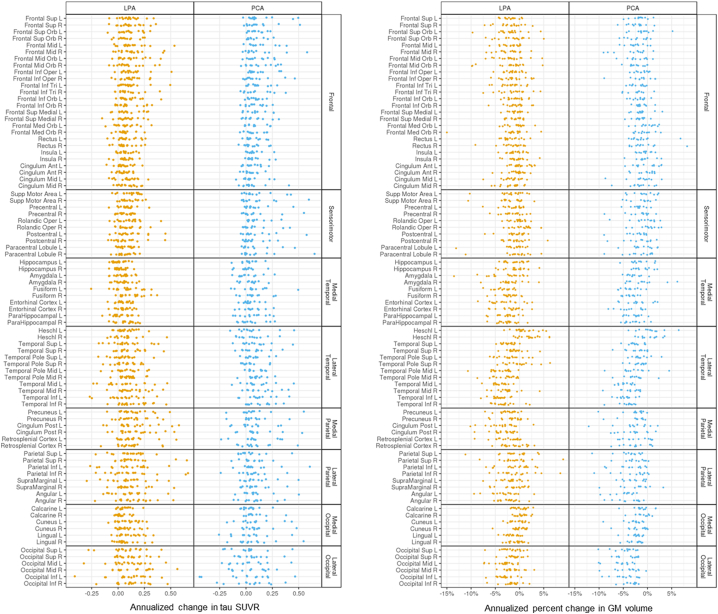
Regional annualized changes in tau SUVR (left) and regional annualized percent changes in MRI grey matter (GM) volumes (right) for each patient.

Results in Figs. 1 and 2 are reported as quantiles of a posterior sample of approximately 400,000 observations for each parameter. Estimates of annualized tau SUVR changes relative to cognitively unimpaired varied across ROIs in atypical AD, with the smallest changes observed in the hippocampus and greatest changes observed in right middle frontal gyrus, right inferior temporal lobe and right posterior cingulate (SUVR changes >0.14 per year). Similar patterns were observed in both LPA and PCA, although LPA also showed large changes (>0.14) in precuneus, angular gyrus, superior parietal lobe and inferior lateral occipital lobe (Fig. 1, left). A negative effect of age was found in the majority of the ROIs in atypical AD, and within LPA and PCA. The greatest age effects were observed in frontal ROIs, with, for example, every decade resulting in up to 0.08 SUVR (right frontal mid) slower annual rate of tau accumulation in atypical AD, i.e. a 75 year old accumulated tau at an annual rate that is up to 0.08 SUVR slower than the annual rate of a 65 year old (Fig. 1, right). There was strong evidence (p > 0.99) that the medial temporal lobe had the lowest rate of tau accumulation relative to the other cortical lobes in atypical AD (Table 2A). The regional annualized changes in tau SUVR for each patient are reported in Supplemental Fig. 1 (left).

The estimated annualized percent change in MRI grey matter volume was highest in lateral temporal, angular gyrus and lateral occipital regions in atypical AD relative to cognitively unimpaired. The lateral occipital lobe had the highest probabilities (p > 0.99) of experiencing more annual volume loss than the other cortical lobes (Table 2B). Both LPA and PCA showed high rates of atrophy in the lateral temporal lobe, with LPA showing left-sided asymmetry and high rates in left posterior cingulate and fusiform, and PCA showing high rates in angular gyrus and throughout the lateral occipital ROIs (Fig. 2, left). Every decade resulted in up to 0.94% (right inferior parietal) for LPA and 0.85% (right frontal mid) for PCA slower annual rate of atrophy, i.e. older individuals experienced a slower volume loss compared to the younger, with some exceptions, like the hippocampus, for which the age effect was in the opposite direction (Fig. 2, right). The regional annualized percent changes in MRI grey matter volume for each patient are reported in Supplemental Fig. 1 (right).

### 3.2 Voxel-based analyses

**Fig. 3:**
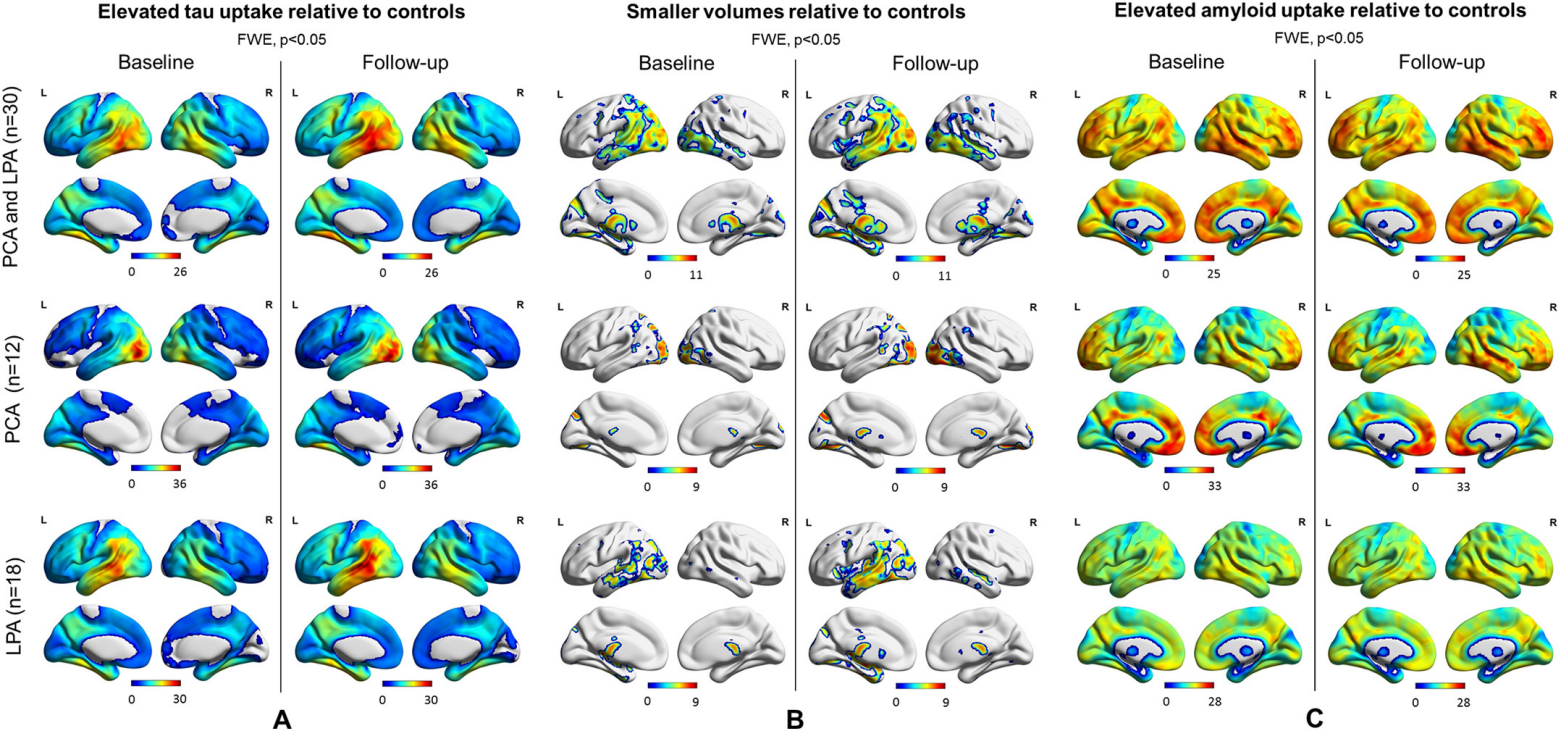
SPM maps of increased tau-PET uptake (A), decreased MRI grey matter volume (B) and increased Aβ-PET uptake (C) at baseline and follow-up for the entire cohort and for the two disease variants relative to cognitively unimpaired. Results are shown after FWE correction for multiple comparison at p < 0.05.

**Fig. 4:**
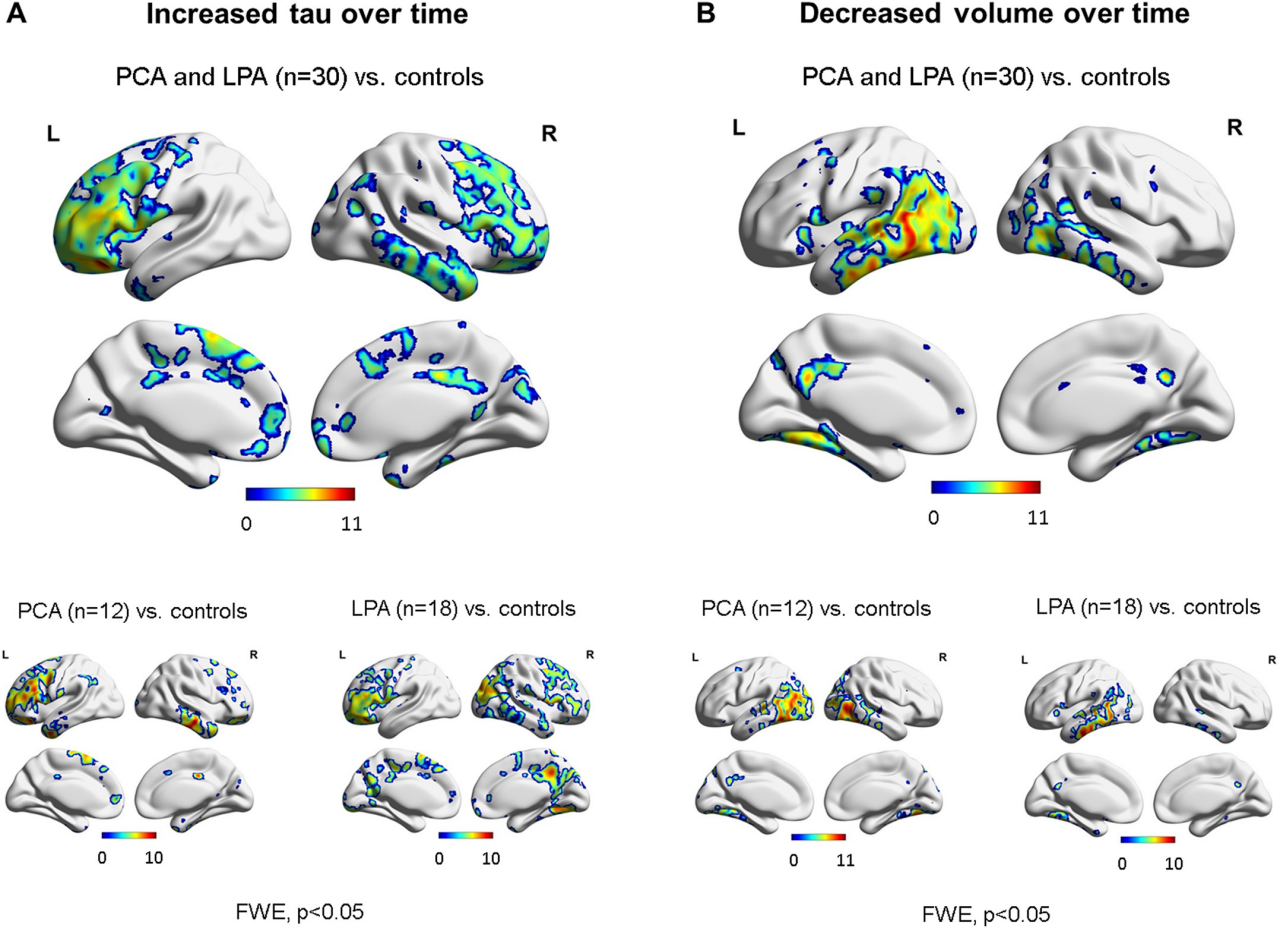
SPM maps of annualized change in tau-PET uptake (A) and MRI annualized log Jacobians (B) for the entire patient cohort (top) and for the two disease variants (bottom) relative to cognitively unimpaired. Results are shown after FWE correction for multiple comparison at p < 0.05.

**Fig. 5:**
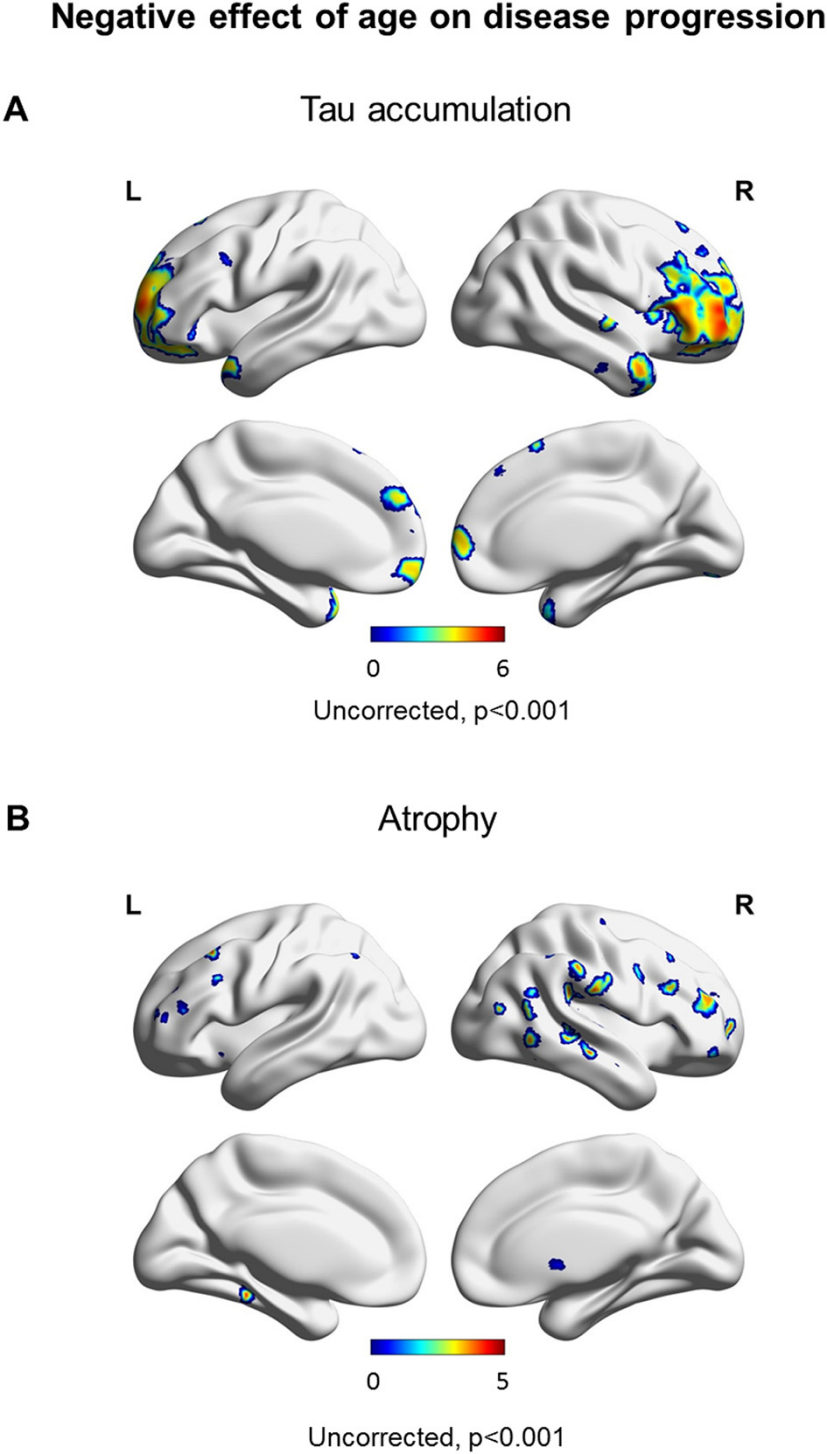
SPM maps of the effect of age on the annualized change in tau-PET uptake (A) and on the MRI annualized log Jacobians (B). Younger patients experienced faster tau accumulation and volume loss than older patients. Results are shown uncorrected at p < 0.001.

**Supplemental Fig. 2:**
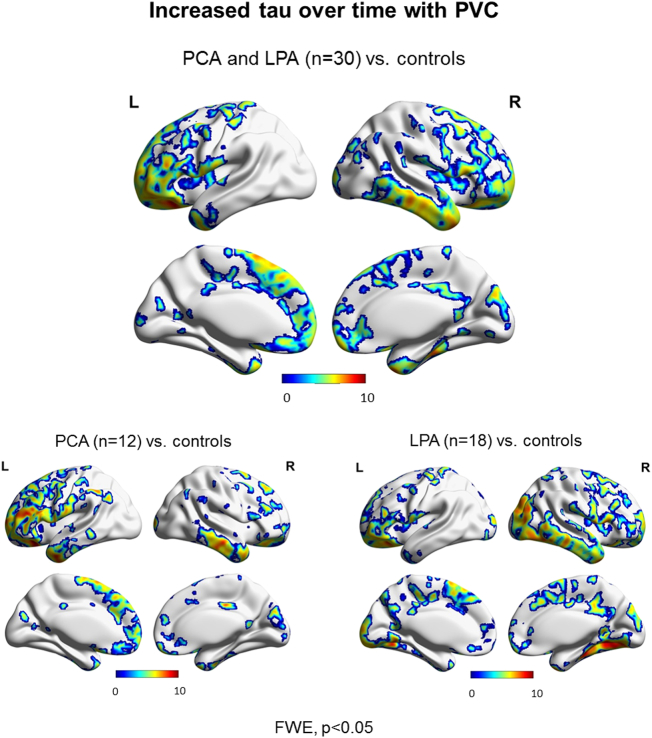
SPM maps of annualized change in tau-PET uptake from partial-volume corrected images for the entire patient cohort (top) and for the two disease variants (bottom) relative to cognitively unimpaired. Results are shown after FWE correction for multiple comparison at p < 0.05.

SPM maps of the baseline and follow-up patterns of tau uptake, volume loss and Aβ uptake are shown after FWE correction for multiple comparisons (p < 0.05) in Fig. 3. Increased tau uptake was observed in the posterior temporal, inferior and medial parietal and occipital lobes, greatest in the left hemisphere, with milder uptake in frontal lobes, in the atypical AD cohort relative to cognitively unimpaired individuals at both baseline and follow-up, with greater severity at follow-up (Fig. 3A). Tau uptake was observed predominantly in the occipital lobes in PCA and in the left temporoparietal lobe in LPA, with LPA showing a greater increase in severity over time. Volume loss presented analogous patterns (Fig. 3B). Aβ uptake was widespread in both PCA and LPA groups relative to cognitively unimpaired at baseline and follow-up (Fig. 3C). SPM maps of longitudinal increased tau accumulation and atrophy in atypical AD relative to cognitively unimpaired are shown after FWE correction for multiple comparisons at p < 0.05 (Fig. 4). No significant regions of change over time were observed in Aβ uptake in atypical AD patients compared to cognitively unimpaired individuals. Increases in tau uptake over time were observed mainly in the frontal lobes bilaterally, with other regions of increase observed in the sensorimotor cortex bilaterally and the right lateral temporal, inferior parietal, medial parietal and occipital lobe in atypical AD (Fig. 4A top). Additionally, no decrease in tau uptake over time was noticed, except for few voxels around the ventricles which can be dismissed as artifacts. In contrast, atrophy was observed mostly in the temporal and parietal lobes bilaterally, with little change observed in the frontal lobes in atypical AD (Fig. 4B top). The analysis in the opposite direction (i.e. increasing volume) did not reveal any findings, except, again, for few voxels around the ventricles. Spatial distributions of atrophy and tau accumulation overlapped partially in the frontal and temporal lobes. Maps of change for the PCA and LPA groups separately are also shown in Fig. 4 after FWE correction for multiple comparison at p < 0.05. PCA patients showed increased tau accumulation mostly in the left frontal lobe, with some findings in the lateral temporal regions (Fig. 4A bottom), and atrophy bilaterally in the lateral occipital and parietal lobes (Fig. 4B bottom). LPA patients showed increased tau accumulation throughout the frontal lobes and also in the right occipitotemporal and medial parietal cortex (Fig. 4A bottom) and atrophy in the left lateral temporal lobe (Fig. 4B bottom). A version of Fig. 4A made with partial volume corrected tau-PET images is available as supplemental material and it shows that the use of partial volume correction did not substantially change the patterns of longitudinal tau uptake (Supplemental Fig. 2). Voxel-based analyses reiterated the age effect on tau accumulation and atrophy already pointed out by the Bayesian hierarchical models, with younger patients declining faster (Fig. 5). The age effect was predominant in the frontal lobes for tau accumulation (Fig. 5A) and in the frontal, sensorimotor and temporoparietal cortex for atrophy (Fig. 5B). With a threshold of p < 0.001, no age effect was noticed in the opposite direction (i.e. older patients declining faster) for neither tau nor volume in any area of the brain.

### 3.3 Multimodal analyses

**Fig. 6:**
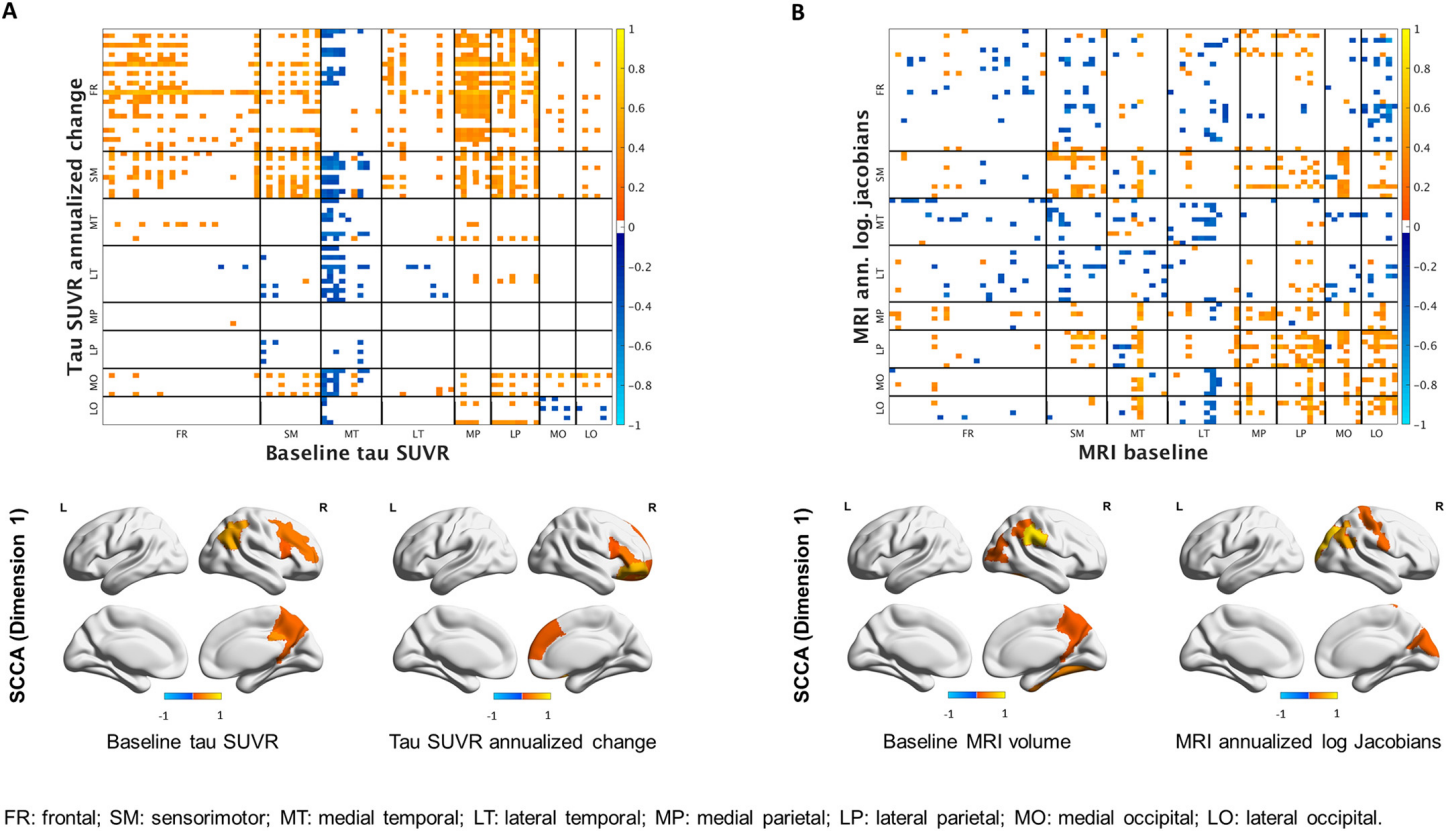
Pearson's correlations (top) and SCCA (bottom) between baseline tau SUVR and tau SUVR annualized changes (A) and between baseline MRI volumes and MRI annualized log Jacobians (B).

Univariate and multivariate analyses revealed two opposite associations between tau SUVR at baseline and tau SUVR annualized change (Fig. 6A). A positive relationship (in red on the heat map) was present, with high tau uptake at baseline across the cortex, particularly in frontal and parietal ROIs, corresponding to high annualized rate of tau accumulation in the frontal and sensorimotor regions (SCCA dimension 1) as well as in the medial occipital ROIs (Fig. 6A). A negative relationship (in blue on the heat map) was also present, with low tau uptake at baseline in the medial temporal lobe corresponding to high annualized rate of change across the brain, particularly in the frontal, sensorimotor, temporal and medial occipital ROIs. For MRI volumes, the relationships were less strong (Fig. 6B) and univariate and multivariate analyses highlighted a local positive association between baseline values and annualized log Jacobians in the occipital and parietal regions (SCCA dimension 1), i.e. regions with already reduced volume at baseline experienced even more atrophy and vice versa (Fig. 6B).

**Table 2:** Between-lobes comparisons of annualized rates of change in tau SUVR (A) and MRI annualized log Jacobians (B). The value in each cell represents the posterior probability that the column label lobe has a higher annualized change than the row label lobe. For example, from A, the frontal lobe is slightly more likely (p = 0.648) to have more tau accumulation than the sensorimotor lobe. From B, the frontal lobe is highly likely (p = 0.993) to have more atrophy than the sensorimotor lobe.

Lobe	Frontal	Sensorimotor	Medial temporal	Lateral temporal	Medial parietal	Lateral parietal	Medial occipital	Lateral occipital
Annualized rates of change in tau SUVR								
Frontal		0.352	<0.001	0.180	>0.999	0.766	<0.001	0.001
Sensorimotor	0.648		<0.001	0.338	>0.999	0.820	<0.001	0.005
Medial temporal	>0.999	>0.999		>0.999	>0.999	>0.999	>0.999	>0.999
Lateral temporal	0.820	0.662	<0.001		>0.999	0.910	<0.001	0.010
Medial parietal	<0.001	<0.001	<0.001	<0.001		0.005	<0.001	<0.001
Lateral parietal	0.234	0.180	<0.001	0.090	0.995		<0.001	<0.001
Medial occipital	>0.999	>0.999	<0.001	>0.999	>0.999	>0.999		0.815
Lateral occipital	0.999	0.995	<0.001	0.990	>0.999	>0.999	0.185	

MRI annualized log Jacobians								
Frontal		0.007	>0.999	>0.999	>0.999	>0.999	0.075	>0.999
Sensorimotor	0.993		>0.999	>0.999	>0.999	>0.999	0.690	>0.999
Medial Temporal	<0.001	<0.001		0.136	0.938	0.652	<0.001	>0.999
Lateral Temporal	<0.001	<0.001	0.864		0.994	0.925	<0.001	>0.999
Medial Parietal	<0.001	<0.001	0.062	0.006		0.130	<0.001	>0.999
Lateral Parietal	<0.001	<0.001	0.348	0.075	0.870		<0.001	>0.999
Medial Occipital	0.925	0.310	>0.999	>0.999	>0.999	>0.999		>0.999
Lateral Occipital	<0.001	<0.001	<0.001	<0.001	<0.001	<0.001	<0.001

**Fig. 7:**
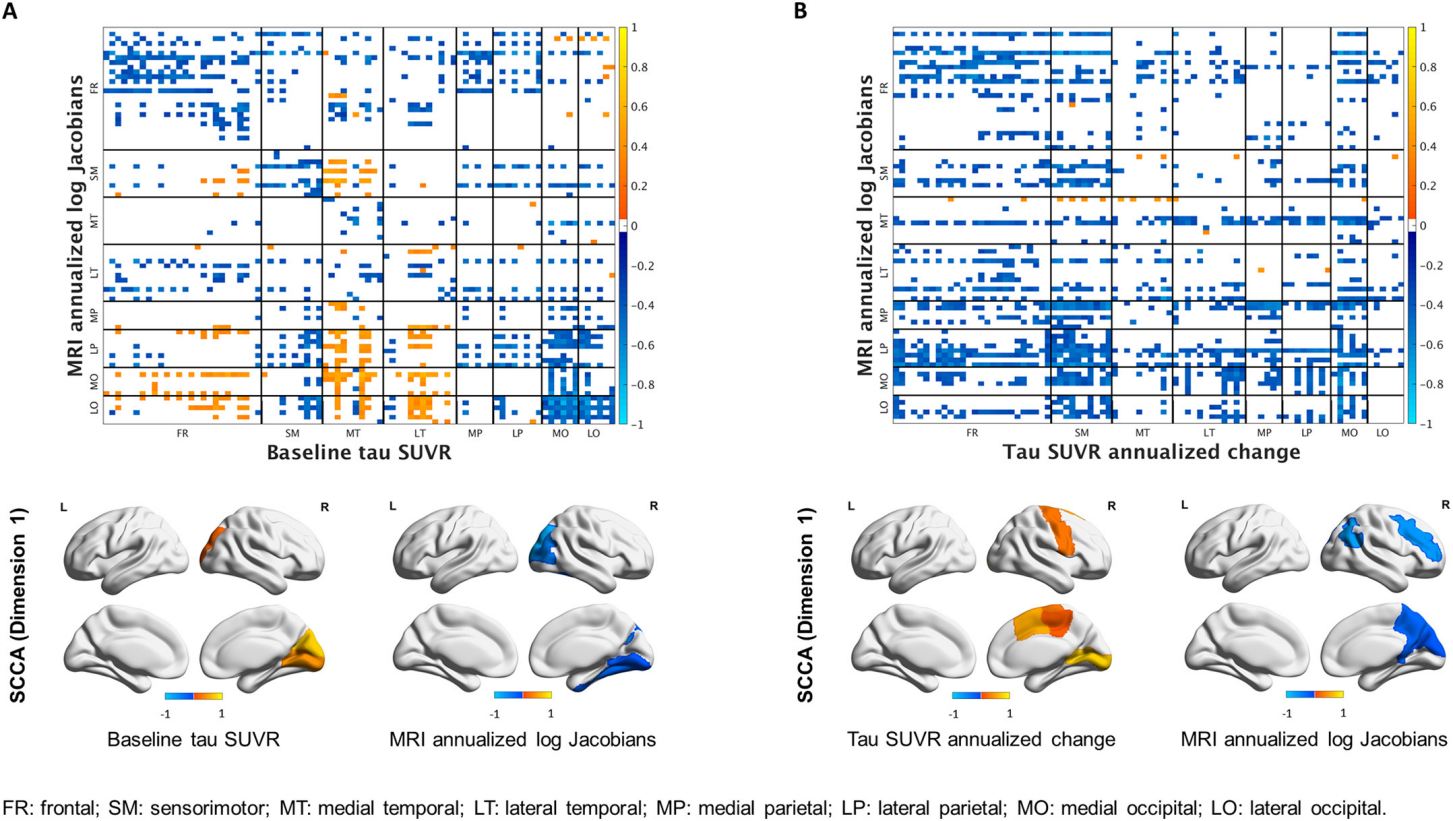
Pearson's correlations (top) and SCCA (bottom) between baseline tau SUVR and MRI annualized log Jacobians (A) and between tau SUVR annualized changes and MRI annualized log Jacobians (B).

Two patterns were present in the multimodal analyses between tau SUVR at baseline and MRI annualized log Jacobians. Strong negative local correlations were observed particularly in the frontal lobe and occipitoparietal cortex, with greater tau SUVR at baseline associated with greater rates of atrophy within these regions (SCCA dimension 1, Fig. 7A). An opposite trend was present between lower temporal and frontal tau uptake at baseline and higher occipitoparietal atrophy. Univariate and multivariate analyses exposed several negative local and distant associations between annualized changes in tau SUVR and MRI annualized log Jacobians (Fig. 7B), meaning that higher rates of tau accumulation were associated with higher rates of atrophy. Specifically, sensorimotor and medial occipital tau accumulation was associated with frontal, occipital and parietal atrophy (SCCA dimension 1, Fig. 7B). In the multimodal analyses, the correlations in the expected direction (i.e. higher rate of tau accumulation associated with higher rate of atrophy) were negative because one measurement was positive (tau accumulation) and one was negative (volume reduction).

## 4 Discussion

The longitudinal patterns of dynamic AD biomarkers, like tau deposition and grey matter loss, and their reciprocal relationships offer insight into the biological mechanisms that drive the progression of atypical AD. To investigate how tau pathology and cortical atrophy change over time and are related to each other in atypical AD, we collected tau-PET and MRI scans at baseline and one year follow-up for 30 patients. We observed that tau uptake and atrophy have different but overlapping longitudinal spreading patterns, where tau accumulates more in the frontal lobes while atrophy increases more in temporoparietal regions, relative to cognitively unimpaired. These pathological changes had several local and distant relationships, where, in general, an increase in tau deposition corresponded to a decrease in volume over time. We also observed a negative effect of age on the progression of the disease, with younger patients having higher rates of tau accumulation and atrophy.

The observed range of estimated regional annualized rates of tau accumulation relative to cognitively unimpaired was comparable to the findings of another recent longitudinal study on typical AD patients, where tau deposition was measured as [^18^F]AV-1451 PET uptake (Jack et al., 2018). The medial temporal lobe presented a slower annualized rate of tau accumulation compared to the lateral temporal, frontal, sensorimotor, parietal and occipital regions and had the lowest probability of accumulating tau faster than the other lobes. The medial temporal lobe has also been shown to have lower tau uptake than cortical regions in cross-sectional analyses (Tetzloff et al., 2018), supporting the notion that this area is relatively spared in the atypical AD phenotypes. This was confirmed by our cross-sectional analyses on tau-PET at baseline and follow-up. An unexpected finding was that, while increases in tau occurred across the cortex in atypical AD, they were most pronounced in the frontal lobes; a finding that was observed in both PCA and LPA groups when analyzed separately. Both groups also showed tau accumulation in the right lateral temporal lobes, with LPA showing additional regions of high tau accumulation in the right lateral occipital and medial parietal lobes. In contrast, cross-sectionally, the temporal, parietal and occipital regions presented the highest levels of tau deposition relative to cognitively unimpaired, at both baseline and follow-up, similarly to what other cross-sectional studies have reported for atypical AD (Cho et al., 2017; Scholl et al., 2017; Tetzloff et al., 2018). Our findings fit the idea of a disease spreading from the regions that were most heavily affected at baseline to other regions of the brain. Hence, in LPA, cross-sectional tau uptake was highest in the left temporal lobe but then appears to spread into the frontal lobe, right temporal lobe, parietal and occipital lobes. In PCA, cross-sectional tau uptake was highest in the occipital lobe and posterior regions of the brain and then appears to spread into the anterior frontal and temporal lobes. Indeed, our univariate and multivariate analyses demonstrated that the rate of tau accumulation in frontal regions correlated with baseline tau SUVR in more typical posterior regions of the brain, showing a spatial disconnect between baseline patterns of uptake and regions of active change. It is likely that tau accumulation in the temporal, parietal and occipital lobes occurs in the early stages of atypical AD, when rates for these regions may be the greatest, and then the disease spreads with faster accumulation in the frontal lobes in the phase that we are capturing with our longitudinal study. Many studies have certainly supported the view that neurodegenerative disease spreads through the brain in this manner (Chiotis et al., 2017; Cho et al., 2016; Ishiki et al., 2015). Notably, we did observe some tau uptake in the frontal lobes at baseline and tau accumulation across the cortex, and hence our results are not too different from a recent study on longitudinal tau-PET in typical AD, which confuted the idea that pathological tau burden increases by spreading from one uninvolved area to the next with no accumulation in previously involved areas (Jack et al., 2018).

A different trend was observed for grey matter volume, where the longitudinal patterns of change focused mostly on lateral temporal, parietal and occipital regions, i.e. the regions that also tend to show maximum atrophy cross-sectionally in atypical AD compared to cognitively unimpaired individuals. Similarly to tau accumulation, the two clinical variants showed slightly diversified patterns of atrophy, more pronounced in the lateral occipital lobe bilaterally for PCA, and in the left temporal lobe for LPA. Unlike the regions of high rates of tau accumulation, which were disconnected from the regions with the highest level of tau deposition at baseline and follow-up, the regions of high rates of atrophy in the PCA and LPA groups mirrored the areas with highest grey matter volume loss at baseline and follow-up. Our univariate and multivariate analyses highlighted local regional correlations between baseline volume and rates of atrophy in the occipital and parietal lobes, meaning that regions in these lobes that showed smallest volumes at baseline were also showing the fastest rates of degeneration. It therefore appears as though we are capturing a different, and perhaps earlier, “phase” of neurodegeneration compared to tau accumulation: longitudinal changes in volume are still occurring in the regions that show the most changes at baseline, while tau accumulation shifted to different regions, i.e. the frontal lobe. Similar findings on different longitudinal spreading patterns for tau uptake (more frontal) and atrophy (more posterior) were shown on a cohort of predominantly typical AD patients (Harrison et al., 2018). We and others have previously suggested that there is a temporal lag between the deposition of tau and the subsequent development of neurodegeneration in AD which may explain these findings (Gordon et al., 2018; Jack et al., 2010; Whitwell et al., 2018). Interestingly, we also observed strong local correlations between tau uptake at baseline and subsequent rates of atrophy in the frontal, parietal and occipital lobes, supporting this concept of a temporal lag and providing evidence that tau deposition leads to future atrophy.

It has recently been shown that, while age has a positive effect on the rates of tau accumulation in cognitively unimpaired individuals, leading to greater rates in older subjects, the effect is opposite for cognitively impaired individuals with abnormal amyloid (Jack et al., 2018). Similarly, we observed that younger patients accumulated tau at a faster rate than older patients. This fits with the accepted notion that the disease is more aggressive when contracted earlier in life (van der Flier et al., 2011), and with cross-sectional studies that have shown greater cortical tau uptake in young compared to old onset AD (Cho et al., 2017; Lowe et al., 2018; Scholl et al., 2017; Tetzloff et al., 2018). While there was evidence that age negatively influenced rates of tau accumulation across all regions, the most prominent effect was in the frontal lobe. A milder effect of age was found on rate of atrophy, with younger patients experiencing a faster decline in volume in frontal and temporoparietal regions. One other study has similarly observed faster rates of atrophy in temporoparietal regions in younger compared to older individuals with typical AD (Fiford et al., 2018).

The relationship between the baseline measurement and its rate of change is crucial to understand the biology of the disease and it differed for the two biomarkers. Baseline volume positively correlated to its annualized rate of change in the parietal and occipital regions, without revealing any other striking pattern. On the contrary, two clearly defined trends were present for tau. For most regions, high levels of tau at baseline were associated with a faster rate of change, both locally and distally. However, the medial temporal regions revealed an opposite behavior, with low tau pathology at baseline associated with faster rates of accumulation in the cortex. This is likely driven by the fact that, cross-sectionally, tau uptake in the medial temporal regions is typically lower than in the other cortical regions for atypical AD patients. Regarding distant associations, it has been speculated that longitudinal tau accumulation occurs through the anatomical and functional networks via white matter tracts and synapses rather than through neighboring areas (Ahmed et al., 2014; de Calignon et al., 2012; Liu et al., 2012). Positive distant relationships between baseline tau uptake and its annualized accumulation rate may support this hypothesis. For example, parietal pathology at baseline was positively associated with accumulation in the frontal regions, implying that tau does not necessarily follow a spreading pattern defined by spatial adjacency, as other studies suggested (Cho et al., 2016; Lowe et al., 2018). Local positive relationships between tau uptake at baseline and its annualized rate of change in frontal, sensorimotor and medial occipital regions may discredit the concept that, once tau has accumulated in one location, it moves to the next Braak region and stops accumulating in the earlier area, and is in accordance with prior data (Jack et al., 2018).

Studies have shown that current levels of tau were related to antecedent rates of atrophy in Aβ-positive individuals (Das et al., 2018; Gordon et al., 2018), supporting the linking of tau burden to neurodegeneration. Our results showed that the higher the tau burden at baseline in the frontal, parietal and occipital regions the more atrophy the patients experienced over time in these regions. These local correlations between tau-PET uptake and atrophy may support the predictive capabilities of tau-PET for neurodegeneration and therefore its use for diagnostic purposes.

As others have previously noted (Lehmann et al., 2013), we found similar patterns of cross-sectional Aβ deposition in the two atypical AD variants. We did not, however, find evidence that regional Aβ uptake significantly changed over time in the atypical AD patients compared to cognitively unimpaired individuals. This is relatively consistent with previous literature in typical AD, where global Aβ has been shown to increase over time (Grimmer et al., 2010; Jack et al., 2009; Villemagne et al., 2011), but not to a greater degree than observed in control subjects (Jack et al., 2009; Villemagne et al., 2011). It has been suggested that the Aβ accumulation in AD eventually plateaus (Jack et al., 2013; Villemagne et al., 2013), resulting in slower accumulation at higher SUVRs.

In our cohort, we did not detect any statistically significant correlations between regional tau accumulation or atrophy and changes in global cognition measured with the MoCA, with the exception of a modest correlation (*R* = 0.41, p = 0.03) with atrophy in the left inferior occipital lobe. This could be attributed to the rather limited number of patients in the study, the fact that we merged LPA and PCA in this analysis, or perhaps that the time interval isn't long enough to adequately capture longitudinal clinical change.

We recognize that our study has some limitations. First, due to the only recent availability of tau-PET, the sample size of atypical AD subjects was relatively small (n = 30); although others have presented longitudinal tau-PET findings on similar sample sizes (Harrison et al., 2018; Jack et al., 2018). The size of PCA and LPA subgroups also limited our ability to run the multimodal and the age effect analyses separately in the two variants, although, importantly, our main findings concerning patterns of progression in the whole cohort were mirrored in each variant. Additional limitations concern the fact tau-PET is a recent technology and the best post-processing method for longitudinal images is still open for discussion, including the use of partial volume correction or the definition of reference regions for SUVR (Harrison et al., 2018; Jack et al., 2018; Southekal et al., 2018; Yang et al., 2018). Our PET images were not partial volume corrected in order to keep the measurements relatively independent from the MRI measurements, without introducing artificial associations between the two. However, we show that the patterns of tau accumulation remain relatively unchanged when partial volume correction was applied. We used the cerebellar crus as the reference region to calculate longitudinal tau-PET SUVR as in (Jack et al., 2018). Others have used white matter regions instead (Harrison et al., 2018), which offer the advantage of a more stable signal over time but could lead to worse group separation performance, due to white matter correlation with the target signal.

## 5 Conclusions

In summary, our study exposed significant spatiotemporal relationships between two key dynamic biomarkers of atypical AD: the deposition of tau protein and the reduction of grey matter volume. Baseline tau burden occurred in expected areas based on phenotype but tau accumulation occurs in remote areas. On the contrary, atrophy mapped onto the clinical phenotype of PCA and LPA better than tau longitudinally. These relationships add insight to the pathophysiological evolution of atypical AD and possibly support the use of longitudinal tau-PET measures in future AD clinical trials. The results could also be critical for the development of future tau therapies since targeting exclusively the occipital lobe for PCA and the lateral temporal lobe for LPA would be inadequate for a tau therapy that aims to prevent the accumulation of the protein throughout the brain.

The following are the supplementary data related to this article.
